# Contrasted Reactivity to Oxygen Tensions in *Frankia* sp. Strain CcI3 throughout Nitrogen Fixation and Assimilation

**DOI:** 10.1155/2014/568549

**Published:** 2014-05-28

**Authors:** Faten Ghodhbane-Gtari, Karima Hezbri, Amir Ktari, Imed Sbissi, Nicholas Beauchemin, Maher Gtari, Louis S. Tisa

**Affiliations:** ^1^Laboratoire Microorganismes et Biomolécules Actives, Université Tunis El Manar (FST) and Université Carthage (INSAT), Campus Universitaire, 2092 Tunis, Tunisia; ^2^Department of Molecular, Cellular & Biomedical Sciences, University of New Hampshire, 46 College Road, Durham, NH 03824-2617, USA

## Abstract

Reconciling the irreconcilable is a primary struggle in aerobic nitrogen-fixing bacteria. Although nitrogenase is oxygen and reactive oxygen species-labile, oxygen tension is required to sustain respiration. In the nitrogen-fixing *Frankia*, various strategies have been developed through evolution to control the respiration and nitrogen-fixation balance. Here, we assessed the effect of different oxygen tensions on *Frankia* sp. strain CcI3 growth, vesicle production, and gene expression under different oxygen tensions. Both biomass and vesicle production were correlated with elevated oxygen levels under both nitrogen-replete and nitrogen-deficient conditions. The mRNA levels for the nitrogenase structural genes (*nif*HDK) were high under hypoxic and hyperoxic conditions compared to oxic conditions. The mRNA level for the hopanoid biosynthesis genes (*sqh*C and *hpn*C) was also elevated under hyperoxic conditions suggesting an increase in the vesicle envelope. Under nitrogen-deficient conditions, the *hup*2 mRNA levels increased with hyperoxic environment, while *hup*1 mRNA levels remained relatively constant. Taken together, these results indicate that *Frankia* protects nitrogenase by the use of multiple mechanisms including the vesicle-hopanoid barrier and increased respiratory protection.

## 1. Introduction

The genus* Frankia* is comprised of nitrogen-fixing actinobacteria that are able to establish a mutualistic symbiosis with a variety of dicotyledonous host plants that results in the establishment of a root nodule structure [1–6]. The bacteria nourish their host plant with combined nitrogen and the plants provide in return carbon and energy. This symbiosis allows actinorhizal host plants to colonize nutrient-poor soils. Besides its life style within the host plant, these bacteria are members of soil community although less information is known about this life style [7]. Under arid tropic and subtropic conditions of North Africa, actinorhizal plants are essentially represented by fast growing and highly tolerant trees from the family Casuarinaceae [8].

Under atmospheric oxygen conditions,* Frankia* actively fixes dinitrogen to ammonium within the root nodules of the host plants and aerobically in culture [9–15]. The oxygen-labile nitrogenase enzyme is localized within specialized thick-walled structures, termed vesicles that are formed* in planta* and* in vitro* [2, 16–18]. Their shape is strain dependent and host-plant-influenced. Vesicles act as specialized structures for the nitrogen fixation process and are formed terminally on short side branches of hyphae that have a septum near their base. The mature vesicle is surrounded by an envelope that extends down the stalk of the vesicle past the basal septum, which separates the vesicle from the hypha. The envelope surrounding the vesicle is composed of multilaminated lipid layers containing primarily bacteriohopanetetrol and its derivatives [19–22]. It is believed that this lipid envelope acts as an oxygen diffusion barrier to protect the nitrogenase enzyme from oxygen inactivation [19].

Unlike other actinorhizal plants,* Frankia* found within the root nodules of* Casuarina* and* Allocasuarina* plants are devoid of symbiotic vesicle structures [23, 24]. A positive correlation was observed between the differentiation of intracellular hyphae and the lignifications of the host-infected cell walls [23]. In several actinorhizal nodules, a low oxygen tension was shown to be consistent with the high concentrations of hemoglobin [2].* Frankia* are known to produce truncated hemoglobins [25–27]. Besides hemoglobins,* Frankia* possess hydrogenases that may act as oxygen-scavenging enzymes [28]. Sequencing of several* Frankia* genomes [29–34] has provided insight on the physiology and opened up new genomics tools for these microbes. These databases have been used in transcriptomics [35–37] and proteomics studies [38–40] on these bacteria. The aim of the present study was to investigate the expression levels for several selected genes involved under different oxygen concentration for the* Casuarina* compatible* Frankia* sp. strain CcI3. These genes were involved in the following functions: nitrogen fixation and assimilation, hopanoid biosynthesis, hydrogen uptake, and oxidative stress.

## 2. Materials and Methods

### 2.1. Culture Conditions and Experimental Design

*Frankia* sp. strain CcI3 [41] was grown and maintained at 28°C in basal MP growth medium with 5.0 mM propionate and 5.0 mM NH_4_Cl as carbon and nitrogen sources, respectively, as described previously [42].

In all experimental procedures,* Frankia* cells were grown for 7 days in 250 mL cylindrical bottles with a working MP medium volume of 50 mL with and without NH_4_Cl for nitrogen-deficient and nitrogen-replete conditions, respectively. Three sets of oxygen tensions were considered: oxic (atmospheric condition), hypoxic (reduced partial pressure of oxygen), and hyperoxic (elevated oxygen levels). Hypoxic conditions were generated by placing the cultures in Brewer's jar that contained reduced partial pressures of oxygen by the use of gas packets (BBL GasPak BBL CampyPak System). For this system, water interacts with catalyst in the packet generating a reduced partial pressure of oxygen within the chamber. Hyperoxic conditions were generated by continuously air-sparging the cultures via an aquarium pump.

### 2.2. Growth Assessment and Vesicle Count

For dry weight determinations, cell cultures were collected on tarred membrane filters (type HA, 0.45 um pore size; Millipore Corp.). The filters were placed in a Petri dish over desiccant and dried at 90°C to constant weight [43]. In parallel, protein content was measured. Briefly, cell samples were solubilized by heating for 15 min at 90°C in 1.0 N NaOH and total proteins were measured using BCA method [44].

Vesicle numbers were determined as previously described [45, 46]. Briefly, cells were sonicated for 30 s with a Braun model 350 sonifier under power setting of 3 using microtip probe. This treatment disrupted the mycelia and released vesicles. The numbers of vesicles were counted by using a Petroff-Hausser counting chamber with a phase-contrast microscope at magnification of 400x.

### 2.3. Determination of Ammonia

Ammonium concentration was determined in cell-free media using modified protocol of Berthelot's reagent [47].

### 2.4. RNA Extraction, RT-PCRs, and Q-PCR

For these experiments, all solutions and materials were DEPC-treated to prevent RNA degradation. RNA extractions were performed by the Triton X100 method as previously described [48]. RNA samples were treated with DNase I (New England Biolabs) according to the manufacturer's recommendations. RNA samples were quantified with a Nanodrop 2000c spectrophotometer (Thermo Scientific) and stored at −80°C until use. The cDNA synthesis was performed using hexamer primers, 400 ng RNA and SuperScript III reverse transcriptase (Invitrogen) according to the manufacturer's recommendations. The cDNA was quantified by a Nanodrop 2000c spectrophotometer, diluted to 10 ng/*μ*L working stocks in DNAse-free, RNAse-free H_2_O, and stored at −20°C until use.

*Frankia* gene expression analyses were performed by qRT-PCR using specific primers (Table 1) and SYBR Green PCR Master Mix (Applied Biosystems) as described previously [49]. Briefly, each 25 *μ*L reaction contained 50 ng template cDNA, 300 nM of the forward and reverse primer mix, and SYBR Green PCR Master Mix. Parameters for the Agilent MP3000 were as follows: (1) 95°C for 15 min, (2) 40 cycles of 95°C for 15 s and 60°C for 30 s, and (3) thermal disassociation cycle of 95°C for 60 s, 55°C for 30 s, and incremental increases in temperature to 95°C for 30 s. Reactions were performed in triplicates and the comparative threshold-cycle method was used to quantify gene expression. The results were standardized with* rps*A expression levels. Relative expression (fold changes) was determined by the Pfaffl method [50] with the control as the calibrator. Two biological replicates of the triplicate samples were averaged.

## 3. Results

### 3.1. Growth and Vesicle Production under Different Oxygen Pressures

**Figure 1:**
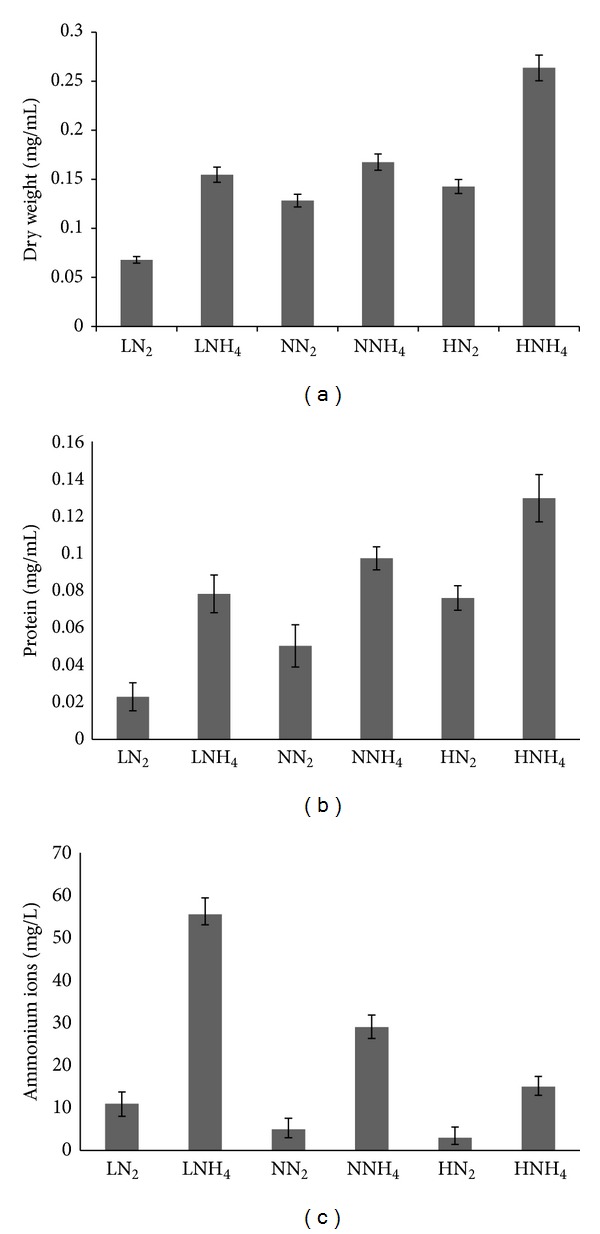
Biomass yields of* Frankia* sp. strain CcI3 grown under nitrogen fixation (N_2_) and nitrogen-replete (NH_4_) at hypoxic (L), oxic (N), and hyperoxic (H) conditions as estimation by (a) dry weight and (b) total protein and determination of (c) ammonium ion concentrations.

Figure 1 shows the effect of oxygen on the growth yield of* Frankia* sp. strain CcI3. Under nitrogen-replete conditions (NH_4_), the biomass of cells grown under hyperoxic conditions was greater than both cultures grown under oxic and hypoxic conditions. Under nitrogen-deficient (N_2_) conditions, the biomass correlated with the oxygen level with the hyperoxic conditions generating the greatest biomass. Furthermore, vesicle production under nitrogen-deficient (N_2_) conditions positively correlated with oxygen tension. Cells under hyperoxic (air-sparged) conditions produced 2.6- and 5.4-fold more vesicles (6.50 ± 0.41 × 10^6^/mg) than oxic (2.45 ±  0.29 × 10^6^/mg) and hypoxic (1.20 ± 0.36 × 10^6^/mg) conditions, respectively. Analysis of ammonia metabolism by* Frankia* CcI3 indicates that it was correlated with oxygen tension. With nitrogen-replete conditions, hyperoxic conditions resulted in the highest ammonia consumption, followed by oxic condition and lastly hypoxic condition (Figure 1(c)). Under nitrogen-deficient conditions the level of ammonium ions increased under lower oxygen tension. This level decreased with corresponding increases in oxygen tension.

### 3.2. Expression of Nitrogen Fixation and Assimilation Genes under Different Oxygen Pressures

**Figure 2:**
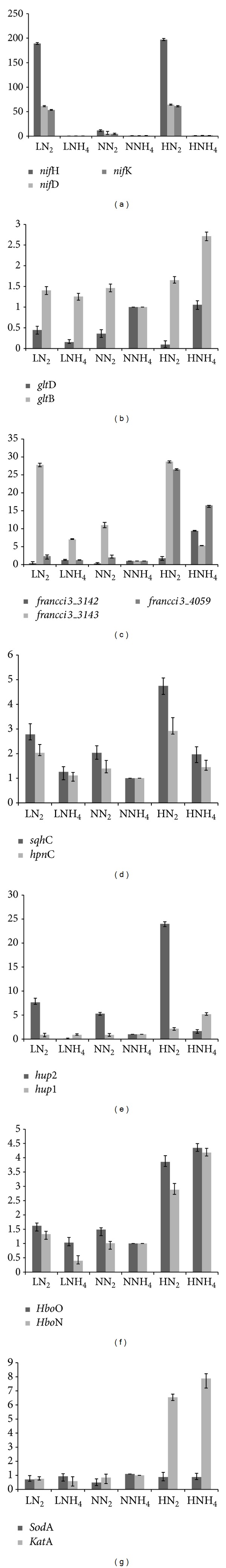
Relative gene expression (fold change) in response to hyperoxic and hypoxic conditions.* Frankia* cultures were grown under nitrogen-replete (NH_4_) or nitrogen-deficient (N_2_) conditions. These cultures were exposed to oxic (N), hyperoxic (H), and hypoxic (L) conditions as described in Section 2. Experimental gene expression was normalized to the rpsA housekeeping gene and compared to the calibrator (NH_4_ oxic conditions). The following genes were analyzed: (a)* nif*HDK (b)* glt*B and* glt*D, (c)* gln*A genes, (d)* hpn*C and* sqh*C, (e)* hup*1 and* hup*2, (f)* hbo*N and* hbo*O, and (g)* sod*A and* kat*A.

The effect of oxygen on the expression of several genes involved in nitrogen fixation and assimilation was measured by detecting changes in mRNA levels via qRT-PCR (Figure 2). For nitrogen-deficient conditions, the level of structural nitrogenase genes (*nif*HDK) mRNA increased >10-fold under hyperoxic and hypoxic conditions compared to oxic condition (Figure 2(a)). Under nitrogen-replete conditions, the expression levels for these genes were very low and there was no change with different oxygen tensions.

The* Frankia* genome contains two glutamate synthase genes (*glt*B and* glt*D) encoding the large and small subunits of the enzyme. These two glutamate synthase genes were studied for their expression levels under three oxygen tensions. The mRNA levels of the* glt*B gene were reduced except under hyperoxic and nitrogen-replete conditions (Figure 2(b)). The* glt*D mRNA levels increased slightly (1.3–2.5-fold) under the different nitrogen and oxygen conditions. There were four glutamine synthetase orthologs found within the* Frankia* sp. strain CcI3 genome. We were able to follow the expression of three of these* gln*A genes (Figure 2(c)). The level of* francci3_3143 *mRNA was controlled by nitrogen. Under all oxygen conditions,* francci3_3143 *mRNA levels increased 10–15-fold under nitrogen-deficient (N_2_) conditions. Both high and low oxygen tensions increased the level of* francci3_3143 *mRNA. The level of* francci3_3142 *mRNA was decreased under nitrogen-deficient (N_2_) conditions and showed 7-fold increase under hyperoxic under nitrogen-replete conditions. The levels of* francci3_4059 *mRNA remained constant except under hyperoxic conditions, in which levels increased 15-fold. Under hyperoxic conditions, the levels of* francci3_4059 *mRNA were controlled by nitrogen status and increased approximately 2-3-fold from nitrogen-replete (NH_4_) conditions.

### 3.3. Expression of Genes Known to Protect Nitrogenase from Oxygen and Reactive Oxygen Species

The biosynthesis of hopanoids has been correlated with vesicle development [19]. The effect of oxygen tension on the expression of the squalene synthase (*hpn*C) and squalene/phytoene cyclase (*sqh*C) genes was examined (Figure 2(d)). Under nitrogen-replete conditions (NH_4_), the level of mRNA for* sqh*C showed a 2-fold increase for hyperoxic conditions. A smaller increase was observed for* hpn*C mRNA levels. In general,* sqh*C and* hpn*C were expressed constitutively with comparable mRNA levels for hypoxic and oxic levels. Under nitrogen-deficient (N_2_) conditions, the mRNA levels of both genes (*sqh*C and* hpn*C) increased 2- and 1.5-fold, respectively.

The* Frankia* CcI3 genome contains two hydrogenase operons [30, 52, 53]. We tested the effects of oxygen tension and nitrogen status of their gene expression levels (Figure 2(e)). Under nitrogen-replete (NH_4_) conditions, the level of mRNA for* hup*2 increased proportionally with the level of oxygen present, while the level of mRNA for* hup*1 only increased under hyperoxic conditions. The expression of* hup*2 was influenced by the nitrogen status of the cells and by the oxygen levels. Under both conditions,* hup*2 mRNA levels increased, but* hup*1 expression remained constant.

The effect of oxygen tension and nitrogen status was investigated on the expression of two truncated hemoglobins (*hbo*O and* hbo*N). The level of mRNA of* hbo*O and* hbo*N increased under hyperoxic condition for both nitrogen conditions (Figure 2(f)). Under nitrogen-replete (NH_4_) conditions, mRNA levels for* hbo*O increased proportionally to the oxygen tension levels. Under hypoxic nitrogen-deficient conditions, mRNA levels for* hbo*N increased about 1.5-fold.

The effects of oxygen tension and nitrogen status on the expression levels of two oxygen defense enzymes, catalase (*kat*A) and superoxide dismutase (*sod*A), were also tested (Figure 2(g)). Under hyperoxic conditions, the mRNA levels of* kat*A increased 6.5- and 8-fold under nitrogen-deficient (N_2_) and nitrogen-replete (NH_4_) conditions, respectively. The expression of the* sod*A gene appeared to be constitutive under all oxygen tensions and both nitrogen statuses.

## 4. Discussion

Without a doubt, the vesicle is the most characteristic morphogenetic structure produced by* Frankia* [1]. Vesicles are functionally analogous to cyanobacterial heterocysts providing unique specialized cells that allow nitrogen fixation under aerobic condition [54, 55]. In this study, the growth of* Frankia* strain CcI3 was evaluated under three oxygen tensions. The results indicate that growth increased with elevated oxygen tensions (Figure 1) confirming the aerobic nature of the microbe. Although the dry weight measurement increased, the total protein values were reduced under hyperoxic nitrogen-deficient (N_2_) conditions. This result would imply that the cells were producing other metabolic products under this condition and a similar level of protein compared to hypoxic nitrogen-deficient (N_2_) condition. Thus, this result suggests that part of the respiration was uncoupled providing some oxygen protection.* Frankia* contains two respiratory systems and a cyanide-insensitive system was proposed to help protect nitrogenase from oxygen inactivation [46]. With other aerobic nitrogen-fixing bacteria, increased respiratory rates in response to elevated oxygen tensions help maintain low levels of intracellular oxygen protecting nitrogenase from inactivation [56, 57]. Under nitrogen-deficient (N_2_) conditions, vesicles were produced and correlated with oxygen tensions. The numbers of vesicles produced per mg dry weight increased with elevated oxygen levels. These results confirm those obtained previously [58, 59].

In our study, we investigated the effects of oxygen on gene expression for a variety of functional genes involved in nitrogen fixation, nitrogen assimilation, and protection from oxygen and other reactive oxygen species [60]. The levels of expression for the structural nitrogenase genes (*nif*HDK) indicate a concordant profile with clear induction under nitrogen-deficient (N_2_) conditions. Transcriptome studies on* Frankia* sp. strain CcI3 under nitrogen-deficient and nitrogen-replete conditions also show an increase in* nif*HDK gene expression [35, 36]. The levels of* nif*HDK mRNA showed an increase under hypoxic and hyperoxic conditions indicating that nitrogenase induction was influenced by oxygen levels.

The hopanoid envelope has been postulated to be involved in the protection of nitrogenase from oxygen inactivation [19]. We found that mRNA levels of squalene synthase (*hpn*C) and squalene-hopene cyclase (*sqh*C) genes increased in response to oxygen tension under nitrogen-deficient conditions, but remained constant under nitrogen-replete conditions (Figure 2(d)). The results correlate with the increase in vesicle envelope observed under high oxygen levels [61]. Nalin et al. [62] found only a slightly higher hopanoid content under nitrogen-deficient conditions suggesting remobilization rather than nascent biosynthesis. Furthermore, the* Frankia* sp. strain CcI3 transcriptome profiles under nitrogen-deficient and nitrogen-replete conditions did not show any significant differences in hopanoid biosynthetic genes [35, 36]. However, these studies were performed under one oxygen tension while our study has investigated three different oxygen tensions.

Analysis of the nitrogen assimilation genes (*glt*B,* glt*D, and* gln*A) is a bit more complex. The* Frankia* CcI3 genome contained several homologues of* gln*A. The mRNA level of* francci3_3143* correlated the best with nitrogen regulation, being increased under nitrogen-deficient conditions. Transcriptome studies have shown that* francci3_3143* expression increased significantly under nitrogen-fixing conditions [35, 36], while all of the other homologues remained consistent. This result would suggest that this gene encoded primary nitrogen scavenging enzyme. The levels of expression were also influenced by elevated oxygen tensions during increased nitrogenase activity. The expression levels of the* glt*B and* glt*D appear to be less influenced by oxygen tension. These effects seemed in agreement with the ammonia metabolism results that showed an increase in consumption under hyperoxic conditions.

Our results on hemoglobin gene expression correlate with previous results [48] that showed no increase in* hbo*N and* hbo*O expression in response to nitrogen status increased under low oxygen tension. However, our results conflict in response to oxygen. We found that both* hbo*N and* hbo*O mRNA levels increased under hyperoxic conditions. The use of the more sensitive qRT-PCR in our study compared to RT-PCR is the best explanation for these differences.

*Frankia* possesses two uptake hydrogenase systems [52, 53]. One of them has been correlated with symbiotic growth and the other to free-living conditions [53]. Our results show that* hup*2 gene expression was influenced by nitrogen status suggesting that it was associated with vesicle production, while* hup*1 gene expression was relatively constant. The levels of* hup*2 mRNA increased proportionally with oxygen tensions suggesting potential oxygen protection mechanism. Anoxic conditions have no effect on hydrogenase gene expression by* Frankia* CcI3 but increased by 30% for* Frankia alni* ACN14a [60]. We did not test anoxic conditions in our study.

Increased oxygen tension can lead to elevated oxidative stress conditions. We investigated the influence of oxygen tensions on reactive oxidative stress genes. While* sod*A expression levels were constitutive,* kat*A gene expression increased under hyperoxic conditions. In general, our results confirm those of Steele and Stowers [63], which examined enzymatic activity levels. They reported an increase in catalase activity in cultures derepressed for nitrogen fixation compared to ammonium-grown cultures.

## Figures and Tables

**Table 1:** Primers used in this study.

Locus tag	Gene	Gene identity	Sequence
*francci3_4488 *	*nif*H	Nitrogenase reductase iron-sulfur protein	5′-CGACAACGACATGAAGACC-3′ 5′-CTTGCCGATGATGCTCTC-3′
*francci3_4487 *	*nif*D	Nitrogenase molybdenum-iron protein alpha chain	5′-AAGGACATCGTCAACATCAGCCAC-3′ 5′-AACTGCATCGCGGCGAAGTTATTC-3′
*francci3_4486 *	*nif*K	Nitrogenase molybdenum-iron protein beta chain	5′-TGACGACGACTCCGGAAACAAACA-3′ 5′-TGTGGTAGACCTCGTCCTTGAACA-3′
*francci3_4496 *	*hup*1	Nickel-dependant hydrogenase, large subunit	5′-AACAAATCTGCGACGTCACGGTCA-3′ 5′-ACTCTCGATCCATTCACCGCAGTA-3′
*francci3_1076 *	*hup*2	Uptake hydrogenase, large subunit	5′-TGGAAGGTCAACTGGCTGGAGAA-3′ 5′-ATGTCTAGGCAGTACCGGAGGAAGAA-3′
*francci3_1149 *	*hbo*O	Truncated hemoglobin	5′-GGGACGCCTGGCTGAAGA-3′ 5′-CCAGAGCTGCCTGTCGAGATC-3′
*francci3_2581 *	*hbo*N	Truncated hemoglobin	5′-CACCCCTCTTTGCCAACCG-3′ 5′-GGTGGTTTCCGTCGGGAC-3′
*francci3_0823 *	*sqh*C	Squalene hopene cyclase	5′-TGCAATGGCTGCTGGACAA-3′ 5′-TGCCGTAGACGTGGTTGAT-3′
*francci3_0819 *	*hpn*C	Squalene synthase	5′-AACTTCCCGGTCTCGCCGTT-3′ 5′-AACGCGTTGAAGTGGAAACGAACC-3′
*francci3_2949 *	*kat*A	Catalase	5′-ACATGCCGGTGTTCTTCATTCAGG-3′ 5′-ACATCATCATGTGGCATCGACTCGG-3′
*francci3_2817 *	*sod*A	Superoxide dismutase	5′-GTGCCAATGACACCCTTGAGAAGA-3′ 5′-AGTGGAGAATATGCCCGGAAAGGT-3′
*francci3_3012 *	*glt*D	Glutamate synthase, small subunit	5′-TGCATGCGACGAACAACTTCCC-3′ 5′-ATGATGCTGACCTCGATCTGCTTG-3′
*francci3_3013 *	*glt*B	Glutamate synthase, large subunit	5′-CGTGCTGAAGGTGATGTCCAAGAT-3′ 5′-AAATAGGCGTCGATCAGTTCCTGG-3′
*francci3_3142 *	*gln*A	Glutamine synthetase, type I	5′-ATGACCCGATCACCAAGGAACAGT-3′ 5′-GGGTTGTAGTCATAACGGACATCG-3′
*francci3_3143 *	*gln*A	Glutamine synthetase, type II	5′-AACTTCTCCACCAGGCAGACGAT-3′ 5′-AGAACTTGTTCCACGGAGCTGTCT-3′
*francci3_4059 *	*gln*A	Glutamine synthetase, catalytic region	5′-TACAACATCGACTACGCGCTTTCC-3′ 5′-ATACCGGAACACGATCTCGAACTG-3′
*francci3_1057 *	*rps*A	30S ribosomal protein S1	5′-CGAAGTCCGTTCCGAGTTC-3′ 5′-CGCCGAAGTTGACGATGG-3′

Locus tag and gene designation were determined from the Integrated Microbial Genomes System (IMG) at the Joint Genome Institute (https://img.jgi.doe.gov/) [51].
